# Association between impaired fasting glycaemia in pediatric obesity and type 2 diabetes in young adulthood

**DOI:** 10.1038/nutd.2016.34

**Published:** 2016-08-22

**Authors:** E Hagman, P Danielsson, L Brandt, A Ekbom, C Marcus

**Affiliations:** 1 Division of Pediatrics, Department of Clinical Science, Intervention and Technology, Karolinska Institutet, Stockholm, Sweden; 2 Unit of Clinical Epidemiology, Department of Medicine/Solna, Karolinska Institutet, Stockholm, Sweden

## Abstract

**Objectives:**

In adults, impaired fasting glycemia (IFG) increases the risk for type 2 diabetes mellitus (T2DM). This study aimed to investigate to which extent children with obesity develop T2DM during early adulthood, and to determine whether IFG and elevated hemoglobin A1c (HbA1c) in obese children are risk markers for early development of T2DM.

**Methods:**

In this prospective cohort study, 1620 subjects from the Swedish Childhood Obesity Treatment Registry – BORIS who were ⩾18 years at follow-up and 8046 individuals in a population-based comparison group, matched on gender age and living area, were included. IFG was defined according to both ADA (cut-off 5.6 mmol l^−1^) and WHO (6.1 mmol l^−1^). Elevated HbA1c was defined according to ADA (cut-off 39 mmol l^−1^). Main outcome was T2DM medication, as a proxy for T2DM. Data on medications were retrieved from a national registry.

**Results:**

The childhood obesity cohort were 24 times more likely to receive T2DM medications in early adulthood compared with the comparison group (95% confidence interval (CI): 12.52–46). WHO-defined IFG predicted future use of T2DM medication with an adjusted hazard ratio (HR) of 3.73 (95% CI: 1.87–7.45) compared with those who had fasting glucose levels <5.6 mmol l^−1^. A fasting glucose level of 5.6–6.0 mmol l^−1^, that is, the IFG-interval added by American Diabetes Association (ADA), did not increase the use of T2DM medication more than pediatric obesity itself, adjusted HR=1.72 (0.84–3.52). Elevated levels of HbA1c resulted in an adjusted HR=3.12 (1.50–6.52). More severe degree of obesity also increased the future T2DM risk.

**CONCLUSION:**

IFG according to WHO and elevated HbA1c (39–48 mmol l^−1^), but not the additional fasting glucose interval added by ADA (5.6–6.0 mmol l^−1^), can be considered as prediabetes in the obese pediatric population in Sweden.

## Introduction

Type 2 diabetes mellitus (T2DM) is a complex and multifactorial disease, which may lead to major morbidities and reduced life expectancy.^1^ In adolescents and young adults, type 2 diabetes seems to be a more aggressive disease than in middle age subjects, demonstrated by a poor response to conventional treatment^2^ and a high mortality rate.^3^

Before the onset of T2DM, a period of disturbed glucose homeostasis is often present, referred to as prediabetes. The prediabetic stage known as impaired fasting glycemia (IFG) is characterized by a moderate hyperglycemia during the fasting state, and in adults, is linked to a marked increased in risk of future T2DM.^4^ However, IFG is also a risk factor in itself for cardiovascular disease, cancer and premature death.^5, 6, 7^

In 2003 an American Diabetes Association (ADA) Expert Committee reduced the cut-off point for the definition of IFG to 5.6 mmol l^−1^ (100 mg dl^−1^), but the World Health Organization (WHO) retained the previous definition of IFG, 6.1 mmol l^−1^ (110 mg dl^−1^).^8^ Furthermore, in 2011 ADA introduced a new criterion for the diagnosis of prediabetes: glycated hemoglobin A1c (HbA1c) of 39–48 mmol l^−1^ (5.7–6.4%).^8^ However, no specific cut-off values for IFG or HbA1c in children are available, resulting in adult standards being used across all age groups.

The prevalence of IFG in children and adolescents varies considerably between countries. We have recently reported that the IFG prevalence in the obese pediatric population is more than three times higher in Sweden compared with Germany.^9^ The prevalence in obese adolescents in USA has been shown to be up to 47% according to the ADA criteria, and we have observed similar figures among severely obese adolescents in Sweden.^10, 11^ However, IFG is also prevalent in pre-pubertal obese children.^12^ Factors shown to affect the risk for IFG in the obese young population include gender, age and degree of obesity.^9^

High-fasting glucose in childhood has been associated with increased adult risk of T2DM in a population-based sample,^13^ although other studies have not been able to detect such an association.^14, 15^ If IFG (ADA) is present in childhood, the risk for remaining IFG or developing T2DM in adulthood is reported to be high.^16^ Whether IFG or prediabetic levels of HbA1c in obese children are additional risk factors over high body mass index (BMI),^13, 14, 15, 17^ for the development of adult T2DM has not been determined, making it unclear, whether prediabetes should be screened for and treated in this population.

We have prospectively studied a large multi-center cohort of children undergoing obesity treatment in Sweden and matched them to a population-based comparison group in regard to gender, age and living area. We have followed them into young adulthood and used collected prescribed anti-diabetic medications as a proxy for T2DM diagnosis.

### Aims

Our aim was to investigate to which extent children with obesity develop T2DM during early adulthood, and to determine whether IFG in obese children increase the risk of developing T2DM. In addition, we aimed to determine whether prediabetic levels HbA1c, the degree of obesity and treatment efficacy in childhood affect the risk for T2DM medication usage in young adults.

## Materials and methods

### Subjects

In this prospective cohort study, the obese cohort consists of subjects included in the national registry for treatment of childhood obesity—BORIS (www.e-boris.se), from March 1995 until April 2013. Inclusion criteria were age 5.0–17.9 years and obese, according to Cole *et al.*,^18^ at the first visit that a fasting glucose measurement were performed. Exclusion criteria were obesity syndromes (Laurence-Moon-Biedl-Bardet, Prader–Willi syndrome and Down syndrome). From the Swedish Total Population Register, a comparison group representing all regions of Sweden was matched by Statistics Sweden. Density matching without replacement was performed in regard to gender, year of birth and geographical living district at the time of obesity treatment using SAS Statistical software (SAS Institute, Cary, NC, USA). No data on blood glucose levels were available for the comparison group. (Syndromes were also excluded from the comparison group *n*=11). One subject from the obese cohort and five subjects from the comparison group were deceased before the age of 18 years and were therefore excluded.

**Figure 1:**
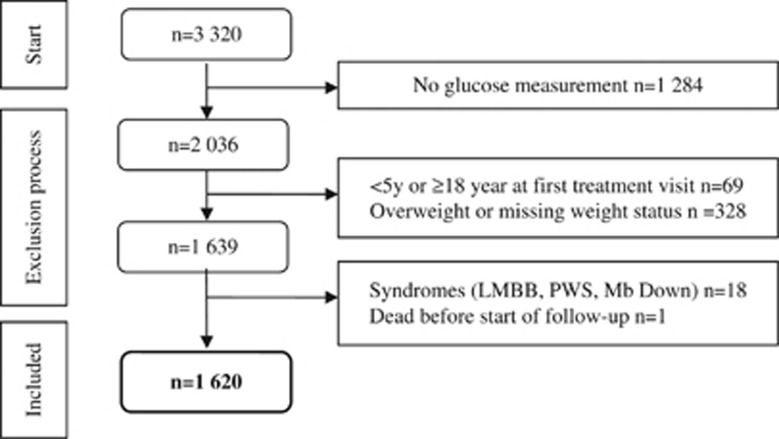
Flow-chart of exclusion process.

In total, 1620 individuals were included in the obese cohort and 8046 individuals in the comparison group, corresponding to five controls for each case. See flow-chart in Figure 1.

All residents in Sweden are assigned a unique personal identity number, which was used for register linkage of the study population by Statistics Sweden. Data on prescribed and collected medications were retrieved from the National Prescribed Medication Registry, which started in July 2005. Data regarding excluded syndromes and deceased individuals were collected from the Patient Registry and the Death Registry. Data on ethnicity were collected from Statistics Sweden.

The most commonly used medication for T2DM treatment is metformin. Exclusive use of insulin is not recommended for new onset of T2DM, except during the first acute phase.^19, 20^ In young adults in Sweden diagnosed with T2DM, 77% receive some kind of pharmacological treatment, whereas 23% receive only diet and exercise advice.^21^ This makes it possible to use anti-diabetic T2DM medications as a marker of T2DM in young adults in Sweden, which is used as the main outcome of this study.

All families have accepted to be registered in BORIS and the study was approved by the Ethics Committee of Stockholm, Sweden (No. 2011/632-31/4).

### Definitions

The first, registered glucose measurement was used to identify IFG using both the ADA definition of ⩾5.6 mmol l^−1^ and the WHO definition of ⩾6.1 mmol l^−1^.^8^ Normal fasting glycemia (NFG) were defined as ⩽5.5 mmol l^−1^. Isolated IFG according to ADA (i-ADA) were defined as 5.6–6.0 mmol l^−1^.

HbA1c value, at the same occasion as fasting glucose value, was diagnosed as prediabetic according to ADA recommendation with a cut-off of 39 mmol l^−1^ (5.7%).^8^

Anti-diabetic medications were defined according to the Anatomic Therapeutic Chemical (ATC) classification system,^22^ where the subgroups of anti-diabetic medications, insulins and analogs (ATC A10A), and blood glucose lowering medications, excluding insulins (ATC A10B) were applied. ATC A10B are henceforth referred to as T2DM medication. The first collected prescribed anti-diabetic medication from 18 years of age was used as a proxy for diabetes.

A Swedish BMI standardized age- and sex-dependent deviation score (the BMI SDS) was used to measure the degree of obesity.^23^

Regarding ethnicity, the subjects were divided into two groups based on countries of birth of both the subjects and their parents: Scandinavian—subjects born in Scandinavia with one or two parents born in Scandinavia, and Non- Scandinavian—subjects born outside Scandinavia or born in Scandinavia with two parents born outside Scandinavia.

Treatment efficacy, expressed as change in BMI SDS, was calculated using the first and last registered BMI SDS in BORIS. Early dropout was defined as children being in treatment <12 months.

### Statistics

SAS Statistical software version 9.4 was used. Kaplan–Meier analysis and Cox proportional hazards regression were used to investigate total medication use related to prediabetes and treatment efficacy. Analysis incorporated adjustment for gender and ethnicity, adding the degree of obesity during childhood/adolescence when analyzing the obese cohort alone. Analysis of proportions between groups were performed using the χ^2^ test. Statistically significant level was set at *P*<0.05 or 95% confidence limits.

## Results

### Characteristics

In total, 1620 obese subjects and 8046-matched comparison subjects were included. The proportion of Scandinavian subjects differed slightly between groups. In the obese cohort, 24.8% were of non-Scandinavian origin compared with 22% in the comparison group baseline characteristics for the obese cohort are presented in Table 1. Median age at end follow-up was 21.1 (range: 18–34.4) years. Data on HbA1c were available in the 71% in the obese cohort. Neither age, degree of obesity or gender differed between those with and without HbA1c data (data not shown).

### Comparison between the groups

The proportion of subjects prescribed insulin or insulin analogs alone (ATC code A10A), indicating type 1 diabetes, was 0.1% (*n*=2) in the obese cohort and 0.7% (*n*=54) in the comparison group (*P*<0.01). T2DM medications (A10B), indicating T2DM, with or without insulin, were collected by 3.2% (*n*=52) in the obese cohort and 0.1% (*n*=11) in the comparison group (*P*<0.0001). Of those subjects with T2DM medication, combination treatment with insulin was evident in 25% in the obese cohort and 73% in the comparison group (*P*<0.0001).

Cox regression analysis, adjusted for gender and ethnicity, demonstrated that the use of T2DM medications differed considerably between the groups, with a hazard ratio (HR) of 24 (95% confidence interval (CI): 12.52–46) (*P*<0.0001). When comparing fasting normoglycemic (<5.6 mmol l^−1^) obese subjects with the comparison group, the adjusted HR was 18.49 (95% CI: 9.29–36.80) (*P*<0.0001). The corresponding analysis for normal HbA1c (<39 mmol l^−1^) was HR 16.59 (95% CI: 8.04–34.22) (*P*<0.0001). Neither gender nor ethnicity affected risk of T2DM medication usage.

### The impact of prediabetes in the obese cohort

The proportions of subjects collecting T2DM medications were 0.1% in the comparison group, 2.4% in the obese NFG group, 4.6% in the obese IFG i-ADA group (5.6–6.0 mmol l^−1^) and 10.5% in the obese subjects with IFG WHO. In the group with normal HbA1c, 2.2% collected T2DM medication vs 9.9% in the group with prediabetic HbA1c values. The proportion of subjects collecting T2DM medication among subjects who were excluded due to no glucose measurement (2.9%) did not differ from those included in the study (3.2%) (*P*=0.4).

In crude analysis were HR for gender 1.69 (0.96–2.97) (*P*=0.07), non-Scandinavian origin 0.90 (0.49–1.66) (*P*=0.7) and degree of obesity 2.06 (1.30–3.26) (*P*=0.002) for each unit increase.

**Figure 2:**
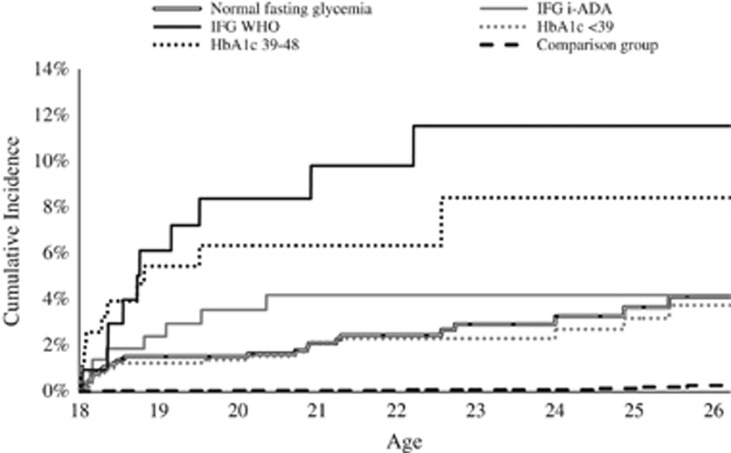
Cumulative incidence of type 2 diabetic medications (ATC A10B) in young adulthood among individuals who have been treated for obesity in childhood. They are divided based on prediabetic levels of fasting glycemia and HbA1c at childhood and compared with a group, matched on gender, age and living area. Numbers to the right in brackets indicate numbers of individuals left in each strain at 26 years of age.

In adjusted models for T2DM medication collection, the HR for i-IFG ADA was 1.72 (95% CI: 0.84–3.52) (*P*=0.14) and for IFG WHO HR=3.73 (95% CI: 1.87–7.45) (*P*=0.0002). Crude analysis showed numbers of the same magnitude. Ethnicity did not affect the outcome in any of the models. Hazard ratios are presented in Table 2. Baseline age did not affect the likelihood of receiving T2DM medication (data not shown). Cumulative incidences of T2DM medication collection are illustrated in Figure 2.

**Figure 3:**
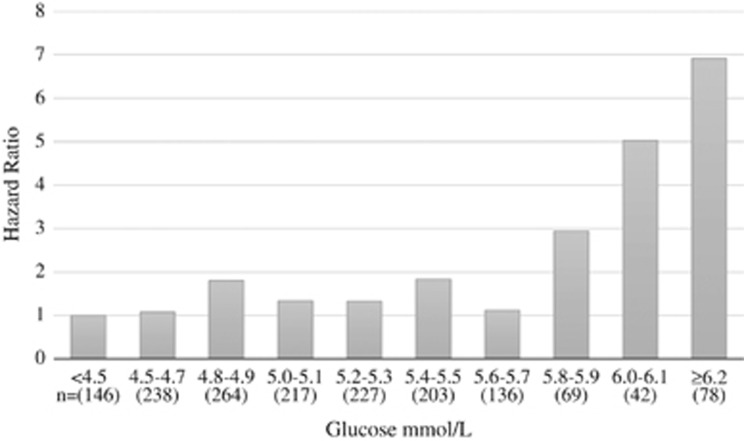
HR for the use of T2DM medication in young adulthood in individuals who have been treated for obesity as children and adolescents. The cohort is divided according to first measured fasting glucose level, and glucose <4.5 mmol l^−1^ is used as a reference. The model is adjusted for gender, degree of obesity and ethnicity.

In an attempt to identify risk thresholds other than established IFG levels, adjusted HR for T2DM medication in different fasting glucose intervals were estimated. Equal risks were observed for all glucose levels <5.8 mmol l^−1^. Statistical significant higher HR, compared with the reference level (<4.5 mmol l^−1^) was found for fasting glucose levels of ⩾6 mmol l^−1^ (Figure 3).

### The impact of treatment efficacy on development of type 2 diabetes

In total 1167 (72%) subjects had at least 1 year of treatment, with a median (range) treatment duration of 3 (1–11.9) years. The remaining 453 (28%) subjects had <1 year in treatment, and were classified as early dropouts and excluded from the treatment analysis.

In crude analysis treatment efficacy, defined as change in BMI SDS, was negatively associated with T2DM medication in adulthood, HR=1.99 (95% CI: 1.24–3.19) (*P*=0.004) for each unit increase in BMI SDS. This remained in models adjusting for gender, ethnicity, IFG_WHO_ and degree of obesity, HR=1.93 (95% CI: 1.16–3.21) (*P*=0.01). The proportion of subjects collecting T2DM medication in the early dropout group (3.1%) did not differ from subjects who changed their BMI SDS by ±0.49 units (2.7%) *P*=0.7.

## Discussion

This study confirms that the pediatric obese population has a markedly higher prevalence of T2DM in early adulthood in relation to a population-based comparison group, regardless of gender and ethnicity. We have used T2DM medication collection as a proxy for T2DM, as the majority of young adults in Sweden with T2DM have been prescribed such medication.^21^ In obese children and adolescents, the WHO-defined prediabetic stage, IFG, predicted future use of T2DM medication in adults, a fasting glucose of 5.6–6 mmol l^−1^, that is, the IFG glucose interval added by ADA, did not increase the use of T2DM medication in young adults more than pediatric obesity itself. Further, a prediabetic HbA1c level predicted T2DM medication to a slightly lower extend than IFG according to WHO. In addition, we found that severe obesity also was a strong risk factor for the incidence of future T2DM medication collection. Female gender was in some, but not all, analyses associated with greater risk for T2DM medication incidence. Weather this is an effect of true T2DM or that some women are treated with metformin for polycystic ovary syndrome remains unknown.

In the adult population, IFG results in a cumulative incidence of T2DM over 6–9 years has been reported with a range of 29–39%.^24, 25^ In the present study the incidence, based on T2DM medication collection, is 10.5% among individuals with IFG WHO and 4.6% in individuals with IFG ADA. This may indicate that IFG in children and adolescents is associated with lesser risk of T2DM development than it is in adults. However, the present study was based on individuals who had been treated for obesity, and although the treatment results were modest, it is possible that the treatment itself reduced the effect of IFG on the risk of future T2DM medication. On the other hand, the subjects who dropped out from treatment did not differ in the proportion of collected T2DM medication from those with a modest treatment effect (±0.49 BMI SDS units). We also found an effect of treatment outcome, which may support this notion.

Diet and physical activity are of importance for co-morbidities related to obesity, independently of degree of obesity. It is possible that individuals who adopt healthy lifestyle upon advice in early years, experience greater effects on long term T2DM risk than would be anticipated from effects of excess weight itself.

In the present study, obese children and adolescents with fasting glucose within the exclusive IFG ADA range did not demonstrate a significantly higher prevalence of T2D medication collection in adulthood than children who were normoglycemic. There were no signs of a protective effect of low fasting glucose levels, but a steep rise in the prevalence of T2DM medication use for individuals who, as children or adolescents, had fasting glucose levels of 5.8 mmol l^−1^ or higher. It has previously been shown in a pediatric population that an elevated fasting glucose level, in normoglycemic (<5.6 mmol l^−1^) children, is a risk factor for future T2DM.^26^ However, these results are probably not contradictory as we have previously shown that obese children in general have elevated fasting glucose levels compared with normal weight children.^12^ It is therefore possible that obesity and overweight contribute to the observed difference in T2DM risk observed in the population-based study of fasting glucose and future T2DM risk.^26^ Indeed, in the present study, we observed an 18.5 times higher risk for T2DM medication collection among obese children and adolescents with NFG than in the control group.

The degree of obesity in adolescents is also of importance for future diabetes risk.^14^ In the present study, each unit increase in BMI SDS, approximately doubled the risk. It has previously been shown that not only obesity, but also overweight in adolescents without any apparent metabolic disturbances, confers a sixfold increase in risk of T2DM more than 20 years later, compared with normal weight controls.^17^

We have previously shown, that obese boys are slightly more prone to demonstrate IFG, a finding also made from other countries.^9, 27^ Despite this, the present study indicated that women used T2DM medications more than men, which is analogous to earlier studies.^24^

The proportion of subjects in the comparison group who used insulins and insulin analogs without concomitant treatment with T2DM medication, i.e. primarily individuals with T1DM, was 0.67%, a similar proportion to that seen in the population in general in Sweden.^28^ The proportion of individuals with T1DM treatment in the obese cohort was much lower. This is probably because T1DM patients in Sweden are treated by teams specialized in T1DM, as opposed to teams treating childhood obesity.

### Limitations

It is possible that the number of subjects who develop T2DM is both under and over diagnosed. We have used medications as a proxy for diabetes, subjects diagnosed with T2DM and solely treated with behavioral modification are not identified. This group is reported to represent approximately 23% in this age group in Sweden.^21^ However, it is unclear whether the percentage of 77% of T2DM patients receiving pharmacological treatment reported for Sweden as a whole, is also correct for this subgroup. T2DM in the young obese population is occasionally silent,^11^ and they remain unidentified in the present study. It is also possible that medical treatment has been initiated for prediabetes and PCO treatment in some individuals and this confounder can cause an overestimation of the development of T2DM, However, differences in development of T2DM between obese children with IFG ADA, IFG WHO and NFG can neither be explained by the use of metformin for other indications than T2DM nor by unidentified individuals with T2DM without pharmacological treatment. The classification of NFG or IFG was based solely on the first blood glucose measurement, in order to eliminate a possible treatment effect. As the reproducibility of IFG has been shown to be moderate,^29^ it is possible that persistent fasting glucose levels between 5.6 and 6.1 mmol l^−1^ would have been significantly associated with T2DM medication. IFG and T2DM prevalence may also differ between countries and ethical origin.^9, 10, 11, 30^ Another limitation is that we do not have any anthropometrical data on the comparison group or for the obese cohort at follow-up.

## Conclusion

Glucose levels between 5.6 and 6.0 mmol l^−1^ (the additional IFG span of ADA) were not associated with increased use of T2DM medication in young adulthood compared with children and adolescents with obesity who had normal glucose levels. Normoglycemic children and adolescents, treated for obesity, were 18 times more likely to be in receipt of T2DM medication in young adulthood than a comparison group. Severe obesity glucose levels of ⩾6.1 mmol l^−1^ (IFG WHO) and high HbA1C levels markedly increased the risk for T2DM.

## Figures and Tables

**Table 1:** Descriptive statistics of the obese cohort (*n*=1 620)

	*Proportion*	*Median (IQR)*
Age at glucose measurement (years)		14.5 (3.4)
Follow-up duration (years)		7.2 (5.8)
Female gender	48.6%	
BMI SDS		3.3 (0.7)
Fasting plasma glucose (mmol l^−1^)		5.1 (0.7)
Isolated IFG ADA (5.6–6 mmol l^−1^)	13.6%	5.8 (0.2)
IFG WHO ( ⩾6.1 mmol l^−1^)	6.5%	6.3 (0.5)
NFG (⩽5.5 mmol l^−1^)	79.9%	4.9 (0.5)
HbA1c (mmol mol^−1^), *n*=1146		35.4 (4.2)
HbA1c (39–48 mmol mol^−1^)	13.6%	40.6 (2.1)
HbA1c (<39 mmol mol^−1^)	86.4%	35.4 (3.1)

Abbreviations: ADA, American Diabetes Association; BMI SDS, body mass index standard deviation score; HbA1c, hemoglobin A1c; IFG, impaired fasting glycemia; IQR, interquartile range; NFG, Normal fasting glycemia; WHO, World Health Organization.

**Table 2:** HR with 95% CI, for collection of T2DM-specific medications in the obese cohort

	*IFG i-ADA*			*IFG WHO*			*HbA1c prediabetes*		
	*HR*	*95% CI*	P *value*	*HR*	*95% CI*	P *value*	*HR*	*95% CI*	P *value*
Prediabetes	1.72	0.84–3.52	0.14	**3.73**	**1.87–7.45**	**0.0002**	**3.12**	**1.50–6.52**	**0.002**
Girls vs boys	**1.99**	**1.04–3.81**	**0.04**	**2.10**	**1.09–4.04**	**0.03**	1.95	0.95–4	0.07
Non-Scandinavian vs Scandinavian	0.84	0.42–1.68	0.62	1.14	0.56–2.32	0.72	1.05	0.47–2.36	0.90
Degree of obesity	**1.79**	**1.03–3.10**	0.04	**1.93**	**1.16–3.21**	**0.01**	**2.11**	**1.14–3.88**	**0.02**

Abbreviations: CI, confidence interval; HbA1c, hemoglobin A1c; HR, hazard ratio.

The models are adjusted for IFG (i-ADA in model 1 and WHO in model 2, *n*=1 620) and prediabetes according to Hba1c (model 3, *n*=1146), gender, ethnicity and degree of obesity. HR and 95% CI were calculated by the Cox's proportional hazards model. IFG i-ADA refers to 5.6–6.0 mmol l^−1^, WHO refers to fasting glucose level of ⩾6.1 mmol l^−1^, and prediabetic HbA1c refers to HbA1c 39–48 mmol l^−1^ (ADA definition). Bold numbers indicate statistical significance.
